# Cardiac Late Sodium Channel Current Is a Molecular Target for the Sodium/Glucose Cotransporter 2 Inhibitor Empagliflozin

**DOI:** 10.1161/CIRCULATIONAHA.121.053350

**Published:** 2021-06-02

**Authors:** Koenraad Philippaert, Subha Kalyaanamoorthy, Mohammad Fatehi, Wentong Long, Shubham Soni, Nikole J. Byrne, Amy Barr, Jyoti Singh, Jordan Wong, Taylor Palechuk, Chloe Schneider, Ahmed M. Darwesh, Zaid H. Maayah, John M. Seubert, Khaled Barakat, Jason R.B. Dyck, Peter E. Light

**Affiliations:** 1 Alberta Diabetes Institute (K.P., M.F., W.L., A.B., J.S., J.W., T.P., C.S., J.M.S., P.E.L.), University of Alberta, Edmonton, Canada.xs; 2 Department of Pharmacology (K.P., M.F., W.L., A.B., J.S., J.W., T.P., C.S., J.M.S., P.E.L.), University of Alberta, Edmonton, Canada.; 3 Department of Pediatrics (S.S., N.J.B., Z.H.M., J.R.B.D.), University of Alberta, Edmonton, Canada.; 4 Faculty of Medicine and Dentistry (S.K., A.M.D., J.M.S., K.B.), University of Alberta, Edmonton, Canada.; 5 Faculty of Pharmacy and Pharmaceutical Sciences (S.K., A.M.D., J.M.S., K.B.), University of Alberta, Edmonton, Canada.; 6 Li Ka Shing Institute of Virology (K.B.), University of Alberta, Edmonton, Canada.

**Keywords:** cardiac sodium channel, clinical perspective, empagliflozin, heart failure, SGLT2 inhibitors

## Abstract

**Background:**

SGLT2 (sodium/glucose cotransporter 2) inhibitors exert robust cardioprotective effects against heart failure in patients with diabetes, and there is intense interest to identify the underlying molecular mechanisms that afford this protection. Because the induction of the late component of the cardiac sodium channel current (late-*I*_Na_) is involved in the etiology of heart failure, we investigated whether these drugs inhibit late-*I*_Na_.

**Methods:**

Electrophysiological, in silico molecular docking, molecular, calcium imaging, and whole heart perfusion techniques were used to address this question.

**Results:**

The SGLT2 inhibitor empagliflozin reduced late-*I*_Na_ in cardiomyocytes from mice with heart failure and in cardiac Nav1.5 sodium channels containing the long QT syndrome 3 mutations R1623Q or ΔKPQ. Empagliflozin, dapagliflozin, and canagliflozin are all potent and selective inhibitors of H_2_O_2_-induced late-*I*_Na_ (half maximal inhibitory concentration = 0.79, 0.58, and 1.26 µM, respectively) with little effect on peak sodium current. In mouse cardiomyocytes, empagliflozin reduced the incidence of spontaneous calcium transients induced by the late-*I*_Na_ activator veratridine in a similar manner to tetrodotoxin, ranolazine, and lidocaine. The putative binding sites for empagliflozin within Nav1.5 were investigated by simulations of empagliflozin docking to a three-dimensional homology model of human Nav1.5 and point mutagenic approaches. Our results indicate that empagliflozin binds to Nav1.5 in the same region as local anesthetics and ranolazine. In an acute model of myocardial injury, perfusion of isolated mouse hearts with empagliflozin or tetrodotoxin prevented activation of the cardiac NLRP3 (nuclear-binding domain-like receptor 3) inflammasome and improved functional recovery after ischemia.

**Conclusions:**

Our results provide evidence that late-*I*_Na_ may be an important molecular target in the heart for the SGLT2 inhibitors, contributing to their unexpected cardioprotective effects.

## Abstract 2

Supplemental Digital Content is available in the text.

**Clinical Perspective**

**What Is New?**

- The sodium/glucose cotransporter 2 inhibitor class of drug has been shown to be highly protective against heart failure, although the precise cellular mechanisms that underlie this effect remain elusive.
- We have identified the cardiac sodium channel as a novel target for sodium/glucose cotransporter 2 inhibitors.
- Using molecular docking techniques, we conclude that the sodium/glucose cotransporter 2 inhibitor empagliflozin binds to same region of the cardiac sodium channel structure as known sodium channel inhibitors such as lidocaine.

**What Are the Clinical Implications?**

- The cardiac sodium channel may therefore be an important molecular target in the heart for the sodium/glucose cotransporter 2 inhibitors that significantly contributes to their beneficial effects against heart failure.
- These drugs may also display efficacy in certain types of arrhythmias caused by long QT syndrome 3 mutations in the cardiac sodium channel.

Despite intensive efforts to improve therapies, heart failure remains a major cause of mortality and hospitalization worldwide.^1^ Therefore, a greater understanding of the pathophysiology and pharmacology of heart failure is a priority.^2^ SGLT2is (sodium/glucose cotransporter 2 inhibitors), such as empagliflozin, have been developed as antidiabetic drugs to promote urinary excretion of glucose.^3^ Surprisingly, recent large-scale cardiovascular safety trials such as the EMPA-REG OUTCOME (Empagliflozin, Cardiovascular Outcomes, and Mortality in Type 2 Diabetes), CVD-REAL (Comparative Effectiveness of Cardiovascular Outcomes in New Users of SGLT-2 Inhibitors), CANVAS (Canagliflozin Cardiovascular Assessment Study), and DECLARE-TIMI 58 (Dapagliflozin and Cardiovascular Outcomes in Type 2 Diabetes) trials demonstrate that SGLT2is significantly reduce the incidence of heart failure by 30% to 40% in patients with diabetes.^4–8^ Furthermore, results from the EMPEROR-Reduced (Empagliflozin Outcome Trial in Patients With Chronic Heart Failure and a Reduced Ejection Fraction) and DAPA-HF (Dapagliflozin in Patients With Heart Failure and Reduced Ejection Fraction) trials^9–11^ demonstrate that the marked cardioprotective effect of the SGLT2is is preserved in the absence of diabetes, suggesting mechanisms independent of improved glycemic control. Consequently, elucidating the precise molecular mechanisms by which SGLT2is afford such robust protection against heart failure is of great importance. It has been suggested that SGLT2is may confer beneficial hemodynamic effects, resulting in reduced plasma volume and preload.^8,12–16^ However, SGLT2is may also directly affect cardiac tissue, and our group has recently shown that empagliflozin reduces the development of heart failure in nondiabetic mice,^17^ which is associated with inhibition of the cardiac NLRP3 (nuclear-binding domain-like receptor 3) inflammasome.^18^ These effects were observed in an ex vivo model of cardiac injury, indicating that there is a direct effect on cardiac tissue in the absence of any hemodynamic/metabolic alterations or diabetes. Moreover, we determined that the inhibitory effects of empagliflozin on the NLRP3 inflammasome are calcium-dependent.^18^ Taken together, these results suggest that a molecular target in the heart for the actions of the SGLT2is may be upstream of the NLRP3 inflammasome and is involved in promoting calcium-handling abnormalities in heart failure.

Detrimental alterations in sodium and calcium homeostasis contribute to ischemia-reperfusion (IR) injury and the development of heart failure,^19^ representing an attractive therapeutic target.^20^ A major pathway involved is the metabolic imbalance-mediated induction of the late/persistent component of the cardiac voltage-gated sodium channel (late-*I*_Na_)^19,21–23^ and increased NHE1 (sodium-hydrogen exchanger isoform 1) activity.^24^ The excessive sodium influx leads to the induction of reverse-mode NCX1.1 (sodium/calcium exchanger isoform 1.1) activity and consequent calcium loading within cardiomyocytes.^21,22^ In this regard, it has recently been shown that SGLT2is inhibit NHE1 activity.^25,26^

Voltage-gated sodium channels provide a crucial role in the initiation of the action potential in many excitable cell types by allowing fast sodium entry, followed by rapid inactivation.^27,28^ However, under certain acquired or congenital conditions, sodium channels fail to inactivate, leading to the generation of late-*I*_Na_ that can be detrimental to cellular health and function. There is strong evidence that the induction of late-*I*_Na_ is involved in the etiology of heart failure and arrhythmias,^21,29,30^ and drugs that are known to inhibit late-*I*_Na_, such as ranolazine, reduce diastolic calcium loading^31^ in heart failure and long QT syndrome 3 (LQT3) models.^32–35^ Because late-*I*_Na_ is induced only in disease states, it is considered to be a promising target for the treatment of heart failure and arrhythmias.^23,29,36–38^ Moreover, congenital LQT3 mutations in the human *SCN5A* gene encoding the major cardiac sodium channel isoform Nav1.5 lead to induction of late-*I*_Na_, which increases sodium entry and action potential duration that precipitates altered calcium handling, torsades de pointes ventricular arrhythmias, and sudden cardiac death.^39,40^ Of further direct relevance is a recent study showing that empagliflozin ameliorates dysfunctional sodium and calcium homeostasis in cardiomyocytes from diabetic rat hearts in part via inhibition of late-*I*_Na_.^41^

Therefore, the aim of the current study was to determine and fully characterize the effects of SGLT2is on late-*I*_Na_ in several complementary model systems. Herein, we detail our discovery that SGLT2is are potent late-*I*_Na_ inhibitors and are highly selective for late-*I*_Na_ compared with peak sodium current (peak-*I*_Na_). We also identify the putative binding pocket of empagliflozin using molecular dynamic drug docking simulations in a validated homology model of the major cardiac sodium channel isoform Nav1.5. These findings indicate that late-*I*_Na_ may be an important molecular target for SGLT2is in the heart, which has implications for our current understanding of SGLT2i pharmacology in heart failure and may also help explain the effectiveness of this class of drug in reducing sudden cardiac death that has been reported.

## Methods

### Data Availability

The data, analytical methods, and study materials will not be made generally available to other researchers for purposes of reproducing the results or replicating the procedure. However, on reasonable request, we will consider sharing certain materials such as the Nav1.5 homology model or Nav1.5 mutant plasmid constructs to individual laboratories. Please contact the senior author via email directly to make such a request.

### Animal Use Guidelines

All animal procedures were institutionally approved by the University of Alberta Animal Welfare Committee in accordance with the guidelines issued by the Canada Council on Animal Care.

### Transverse Aortic Constriction (TAC) Surgery Model of Heart Failure

Male C57BL/6 N mice (7 weeks of age) were obtained from Charles River Canada (St. Foy, Quebec) and housed in a temperature and humidity-controlled animal facility with a 12:12-hour light-dark cycle for a minimum of 1 week before investigation. Animals were maintained on Rodent Chow #5001 and water ad libitum. Eight-week-old mice weighing 20 to 25 g were anesthetized with a cocktail of ketamine (100 mg/kg) and xylazine (20 mg/kg) intraperitoneal and preoperative analgesia (meloxicam, 1–2 mg/kg subcutaneous). Once animals reached a surgical plane, endotracheal intubation was performed using 22-G tubing. The endotracheal tube was then connected to a ventilator (Hugo-Sachs Electronik Mini-Vent, Harvard Apparatus Canada) and ventilated at 200 to 250 µl stroke volume and 150 breaths/min. Animals were then prepared for surgery (Liquid Tears applied to eyes; chest and throat shaved and washed and topical antiseptic utilized, then draped). A vertical skin incision was made from the suprasternal notch to midsternum, and then straight blunt scissors were used to cut the sternum to the third or fourth rib. Mini-Goldstein (FST) spreaders were used to retract the ribs. The thymus was blunt-dissected and the aortic arch visualized. The transverse arch was carefully dissected free of connective tissue, and a 6/0 silk tie was passed around the arch between the innominate and left carotid arteries. A double-blunted 27-G (0.4-mm) needle was then placed alongside the arch and the tie ligated. The blunted needle was then removed to standardize the stenosis. The thymus was gently replaced, and retractors removed. Two sutures were placed in the sternum, and the skin incision was closed using 6/0 Vicryl suture continuous pattern. Sham-operated animals underwent the same procedure without banding. Animals were removed from the ventilator and left to recover in a prewarmed cage with no bedding until the “righting reflex” was achieved, and checked initially every 5 to 10 minutes for the first 60 minutes, then regularly at 20 to 30 minutes intervals for the next 2 to 3 hours. When the animal had regained consciousness and was moving around, bedding, ad libitum food, and water were added to the cage. The home cage was kept on a temperature gradient heated surface for 2 to 3 days, depending on the type of surgery. Analgesics were given for 72 hours after surgery. To confirm the development of heart failure, 2 weeks after surgery, animals were lightly anesthetized with isoflurane, the chest was shaved, and a 30-MHz Doppler probe from the 3100 Vevo Preclinical Imaging System (FUJIFILM Visual Sonics, Toronto, Canada) was used to measure the pressure gradient (mm Hg) or velocity (mm/s) across the aortic constriction.

### Cardiomyocyte Isolation

Three weeks after surgery, sham and TAC mice were injected with 100 U of heparin before euthanasia with an intraperitoneal injection of euthanyl (120 mg/kg body weight). The heart was surgically removed and clamped to a 21-G cannula via a small section of aorta. The heart was then perfused with Krebs-Henseleit buffer containing (in mmol/L ) 118 NaCl, 4.7 mmol/L KCl, 1.2 mmol/L KH_2_PO_4_, 1.2 mmol/L MgSO_4_.7H_2_O, 2.5 mmol/L CaCl_2_.2H_2_O, 25 mmol/L NaHCO_3_, and 5 mmol/L glucose (pH 7, 37°C, 4 mL/min flow rate). The lungs and thymus tissue surrounding the heart were removed, and 6/0 silk suture tied around the aorta in TAC mice was carefully removed to allow free perfusion of the buffer. The heart was then perfused with calcium-free perfusion buffer for 4 minutes containing (in mmol/L ) 120.4 NaCl, 14.7 KCl, 0.6 KH_2_PO_4_, 0.6 Na_2_HPO_4_, 1.2 MgSO_4_.7H_2_O, 10 Na-HEPES, 4.6 NaHCO_3_, 30 taurine, 10 2,3-butanedione monoxime, and 5.5 glucose (pH 7.0 at 37°C). The heart was digested for 12 to 15 minutes with a perfusion buffer containing Collagenase Type II (Worthington Cat No. CLS-2) and 50 µmol/L CaCl_2_. Ventricles were cut and transferred into Erlenmeyer flasks and digested further for 5 to 7 min with 2× trituration in between to release the cardiomyocytes. After neutralizing the digestion with a stop buffer containing 10% bovine calf serum and 12.5 µmol/L CaCl_2_, the cells were filtered using a 100-µm cell strainer and centrifuged at 20 *g* for 10 min. The cells in the supernatant were then reintroduced with calcium at 100, 400, and 900 µmol/L before use for patch-clamp.

### Sodium Current Recording From Sham and TAC Mouse Cardiomyocytes

The whole-cell patch-clamp technique was used to study macroscopic Na^+^ currents at room temperature (22±2°C). Pipettes were pulled from borosilicate glass (World Precision Instruments, Sarasota, FL) using a P-87 micropipette puller (Sutter Instruments, Novato, CA), and the tips were fire-polished, (yielding resistances of 2 to 4 MΩ), and filled with the following pipette solution (in mmol/L): 5 NaCl, 130 CsCl, 5 EGTA, and 10 HEPES; pH was adjusted to 7.2 with CsOH. Cells were placed in a perfused (2–2.5 mL/min) chamber using extracellular solution containing the following (in mmol/L): 20 NaCl, 120 NMDG (N-Methyl-D-glucamine chloride)-Cl, 3.5 KCl, 1.8 CaCl_2_, 1 MgCl, 10 glucose, and 10 HEPES. pH was adjusted to 7.4 with NaOH. After formation of a GΩ seal, the seal was ruptured using suction to gain access to whole-cell voltage-clamp mode. Series resistance was compensated to 70% to 90%. Currents were sampled at 50 kHz and low-pass-filtered at 20 kHz. Whole-cell sodium currents were recorded by stepping from a holding potential of −120 to 0 mV for 400 ms at a frequency of 1 Hz. Whole-cell currents were recorded, digitized, and stored using an Axopatch 200B amplifier, Digidata 1322A, and pCLAMP 9 (Molecular Devices, Union City, CA). Currents were measured as peak, total (area under the curve), and at 60 ms after peak (for late-*I*_Na_). Analysis was performed using Clampfit 9 (Molecular Devices, Sunnyvale, CA), and subsequent curve-fitting and statistical analysis was undertaken using GraphPad Prism (GraphPad Software, La Jolla, CA).

### Nav1.5 Site-Directed Mutagenesis

The human Nav1.5 gene (SCN5A) in the pcDNA3.1(+)/C-(K)DYK vector (GenScript, Cat No. OHu18270D, USA) was cotransfected with a GFP vector into HEK293T cells using Lipofectamine 3000 (Invitrogen, Cat No. L3000001, USA). Transfected cells were seated on coverslips and used for functional studies within 48 to 72 hours. The single amino acid mutations (F1760L, W1345L, Y1767L) and the ΔKPQ deletion mutation were introduced into the SCN5A gene using the QuikChange Lightning Site-Directed Mutagenesis kit (Agilent, Cat No. 210518, USA). PCR products were cloned into XL-10 Gold Ultracompetent cells (Agilent, Cat No. 200315, USA), and DNA was isolated and purified by GeneElute Plasmid Miniprep kits (Sigma, Cat No. PLN350, USA). The presence of the desired mutations and the integrity of plasmids were then verified by DNA sequencing (The Applied Genomics Core, University of Alberta). The primer sequences used to generate mutants in Nav1.5 are listed in the Table. The R1623Q LQT3 mutation in human Nav1.5 was constructed as described previously.^42^

**Table.**
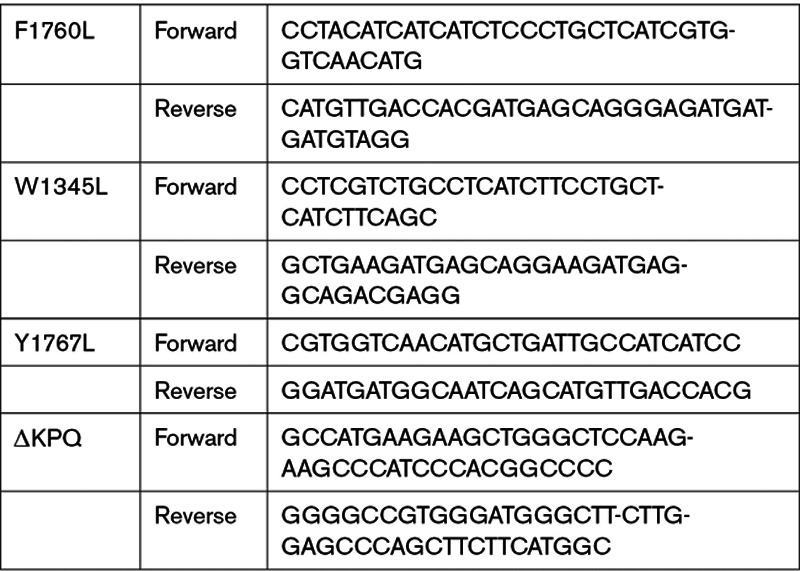
Nav1.5 Mutants Primer Sequences

### Whole-Cell Patch-Clamp Measurement of Recombinant Sodium Current and NCX1

HEK293T cells were cotransfected with expression vectors encoding GFP and human heart Nav1.5 (WT, ΔKPQ, R1623Q, F1760L, Y1767L, or W1345L) or human NCX1.1. Cells were recorded from 24 to 72 hours after transfection as described previously.^42^ Mycoplasma contamination checks were performed routinely, and the HEK293T cell line was found to be negative for mycoplasma. Pipettes were pulled from borosilicate glass (World Precision Instruments, Sarasota, FL) using a P-87 micropipette puller (Sutter Instruments, Novato, CA), and the tips were fire-polished, yielding resistances of 3 to 4 MΩ. The pipette solution contained (in mmol/L) 130 CsCl, 5 NaCl, 5 TEA (tetra-ethyl ammonium chloride)-Cl, 2.5 HEPES, 1 EGTA, and 2 MgATP. The pH was adjusted to 7.2 with CsOH. The extracellular solution contained (in mmol/L) 140 NaCl, 10 HEPES, 1 CaCl_2_, 1.4 MgCl_2_, 5 KCl, and 5 glucose (pH adjusted to 7.4 with NaOH). In some experiments where the effect of glucose on late-*I*_Na_ was examined, glucose concentrations were adjusted accordingly. Solutions were applied to cells using a multi-input perfusion pipette. Whole-cell voltage-clamp was used to record macroscopic peak- and late-*I*_Na_. Data were acquired using an Axopatch 200B patch-clamp amplifier and Clampex 9.2 software (Axon Instruments, Foster City, CA). *I*_Na_ currents were elicited by 300-ms voltage steps to 0 mV from a holding potential of –100 mV with a frequency of 0.2 to 3 Hz. For the measurement of peak- and late-*I*_Na_, only peak currents >2 nA were used for the analysis. Late-*I*_Na_ current was induced by the addition of 30 µmol/L H_2_O_2_. Outward reverse-mode currents generated by NCX1.1 activity were elicited by rapidly switching the solution flowing from a multi-input perfusion pipette from a cesium-based intracellular solution containing (in mmol/L) 120 CsCl, 20 TEA, 5 HEPES, 10 glucose, 2 MgATP, 1.4 MgCl_2_, 4.28 CaCl_2_, and 5 EGTA to a sodium-based intracellular solution containing (in mmol/L) 30 CsCl, 90 NaCl, 20 TEA, 5 HEPES, 10 glucose, 2 MgATP, 1.4 MgCl_2_, 4.28 CaCl_2_, and 5 EGTA. The pH of these solutions was adjusted to 7.2 with CsOH. Only cells with a seal resistance of >1 GΩ were used. All measurements were conducted at room temperature (21–23°C)

### Measurements of Calcium Transients in Single Mouse Cardiac Myocytes

Cardiomyocytes were isolated from 3- to 6-month-old male C57Bl6N mice as described above and then loaded with 1 µmol/L Fluo-4 am for 30 to 60 minutes at room temperature before imaging with an Olympus IX83 inverted microscope. The cells were perfused with a gravity controlled, multi-channel superfusion system at a flow rate of 1.5 mL/min. Fluorescence was recorded at 20 Hz through a UPLSAPO 20X objective and an Andor iXon Ultra 897 camera after excitation at 480 nm for 9.85 ms at 5% intensity of the X-cite 120 LED Boost Excelitas light source. Cardiomyocytes were field-stimulated at 1 Hz at a current value 10% above the threshold current required to elicit contraction using a current stimulator (Warner Instruments, SIU-102) equipped with platinum electrodes submerged in the recording bath. Cardiomyocytes were selected on the basis of their typical rod-shaped morphology, the absence of unpaced calcium events, and their consistent response to field stimulation. Only cells that showed stable calcium transients on pacing were used. Regions of interest were selected such that the cardiomyocyte resided within the boundaries of the regions of interest in both its contracted and its relaxed state. The average fluorescence intensity for the regions of interest in every frame was then analyzed. Fifty-second segments of the fluorescence intensity trace in control and treated conditions from the same cardiomyocyte were obtained. Typically, these segments were the last 50 s during the >120 s superfusion of the chosen treatment condition. The nonevoked calcium events were identified and counted as a local maximum in the calcium trace that did not coincide with a field potential stimulation, and compared the number of unpaced events in control conditions with the number of events during the 20 nmol/L veratridine treatment in the presence or absence of 1 µmol/L empagliflozin.

### Homology Modeling of Human Nav1.5 (hNav1.5) Channel

The amino acid sequence of the full-length human Nav1.5 alpha subunit was obtained from the universal protein resource (UniProt) database (accession ID: Q14524). A sequence similarity search with the human Nav1.5 sequence against the Protein Data Bank database, using the BLASTp^43^ program, identified the human Nav1.4,^44^ at the time of model construction, as the closest available homologous structure with a sequence identity of 66%, with the transmembrane domains in the Nav1.5 and hNav1.4 sharing >75% sequence identity. Therefore, the cryo-electron microscopy structure of human Nav1.4 (Protein Data Bank: 6AGF) in the open state was used as the template to build the homology model of the open human Nav1.5 channel. A homology model of Nav1.5 encompassing 4 transmembrane domains and the extracellular domains were modeled using the knowledge-based method implemented in the Prime module^45^ of the Schrödinger drug discovery suite. Because there was no suitable template available to model the cytoplasmic domains of Nav1.5, these regions were excluded in the model-building step. The extracellular loops in the model were refined using the Prime loop refinement algorithm.^45^ The protonation states of the residues were assigned using the PROPKA program using a pH of 7.0.^45^ The final model was prepared and energy-minimized using the Impref utility in the protein preparation wizard^46^ in Maestro (Schrödinger LLC). The stereo-chemical quality of the prepared model was assessed using a Ramachandran plot,^47^ and the local quality of the model was evaluated using the QMEANBrane program. To further validate our own homology model, we performed a structural alignment between our Nav1.5 homology model and the 2 recently published cryo-EM Nav1.5 structures (Protein Data Bank IDs: 6UZ3 and 6UZ0).^48^ Our new alignment analysis reveals an excellent structural similarity between our model and these 2 cryo-EM structures, with an overall root mean square deviation and percentage identity of 1.77 Å and 91%, respectively (Figure I in the Data Supplement). A close-up on the residues forming the binding site for empagliflozin in Nav1.5 shows almost no translational or rotational differences between our model and the 2 cryo-EM structures (Figure II in the Data Supplement). This new analysis now provides a validation of our model and suggests that none of the tested compounds is expected to show any different binding modes when comparing the newly published cardiac Nav1.5 cryo-EM structures^48^ with our own homology model of Nav1.5 based on the cryo-EM structure of Nav1.4.

### Molecular Docking

Different modules within the Schrödinger drug discovery package were used for binding site identification and molecular docking protocols. Exploration of potential ligand binding cavities in our Nav1.5 model using the SiteMap module^49^ resulted in 9 sites (S1–S9) with a minimum of 15 site points each. Independent receptor grids covering each of these binding sites were generated using the grid generation protocol of the Glide module.^50^ The ligand empagliflozin (CHEMBL2107830) was prepared using the Ligprep module and then docked into the receptor grids on the Nav1.5 model using the Glide extra precision (XP) module.^51^ During the docking process, the van der Waals radii were scaled to 0.8 such that a modest induced-fit effect is introduced into the receptor. A second stage focused docking was carried out within the 5 sites positioned on (1) the central cavity, (2) DI-DII fenestration (f; fDI-DII), (3) DII-DIII fenestration (fDII-DIII), (4) DIII-DIV fenestration (fDIII-DIV), and (5) DI-DIV fenestration (fDI-DIV).

### System Preparation for Classical Molecular Dynamics

The selected poses from molecular docking calculations were subjected to molecular dynamics (MD) simulations under physiological conditions. In each case of the ligand-Nav1.5 complexes, the principal axis of the channel was aligned with the *z* axis and was embedded in a 120×120 Å 1-palmitoyl-2-oleoyl-sn-glycero-3-phosphocholine lipid bilayer using the Membrane plugin of the VMD software.^52^ The membrane-embedded complexes were solvated in a periodic cubic box filled with explicit transferable intermolecular potential with 3 points water molecules (using the Solvate plug-in in VMD), and electro-neutralized with an ionic concentration of 150 mmol/L of NaCl (using the Autoionize plug-in). Structural parameters for the ligands (empagliflozin, dapagliflozin, and canagliflozin) were generated with the Swissparam server.^53^ The parameters for the Nav1.5 protein, lipids, and ions were assigned using the CHARMM36 force field.^54^ All MD simulations were performed using the NAMD (v2.12) molecular dynamics software. Each of the prepared systems was initially energy minimized in 2 stages. During the first stage, the systems were energy-minimized for 250 000 steps by fixing the protein and lipid heads, while allowing movement of the lipid tails. This stage was essential to remove any atom overlaps from improper packing of the membrane around the protein. In the next step, a harmonic restraint of 1 kcal/mol.Å was placed on the protein atoms, and the systems were energy-minimized for 250 000 steps. After the energy minimization, an all-atom equilibration was performed for 5 000 000 steps at a 2-fs time-step. During MD equilibration, the Langevin thermostat^55^ was used to maintain a constant temperature of 310 K and a 1-atm pressure. A 12-Å cutoff with a switching distance and pair-list distance of 10 Å and 13.5 Å, respectively, was used for treating the long-range electrostatics using the Particle Mesh Ewald method.

### Molecular Mechanics Generalized Born Solvation Area Calculation

Binding free energies of the ligand-Nav1.5 complexes were calculated using the MD-based Molecular Mechanics Generalized Born Solvation Area method implemented in the MMPBSA.py script^56^ of the AMBERTools. The GBradii tool was used for estimating the free energies.^57^ The binding free energy for each selected frame was computed using the following equation:


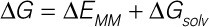


The final binding free energies were averaged over 21 snapshots sampled from the last 8 ns of equilibration trajectories. ParmEd package of AmberTools was used for converting the CHARMM topologies to Amber formats. The per-residue contributions to the total binding free energies were computed using the decomposition protocol implemented in the MMPBSA.py program of AmberTools.

### Mouse Heart Perfusions

All animal protocols were approved by the University of Alberta Animal Care and Use Committee as per institutional guidelines. Hearts were isolated from 12- to 13-week-old male C57Bl/6 mice and perfused in Langendorff mode.^58–60^ Our standard protocol for this type of study is to perform a minimum of 6 or 7 whole heart experiments with strict inclusion criteria based on baseline function (ie, temperature, time to excise the heart from the animal, baseline left-ventricular developed pressure (LVDP), heart rate, and systolic and diastolic function). Only data from hearts that exhibited no arrhythmias and good baseline aerobic function (LVDP >80 cm H_2_O) at the start of each experiment were used. Briefly, mouse hearts were perfused at a constant flow-rate for 40 minutes of baseline stabilization and then subjected to 30 minutes of global no-flow ischemia followed by 40 minutes of reperfusion. Hearts were perfused with vehicle (DMSO), empagliflozin (1 µmol/L), or tetrodotoxin (22.5 µmol/L). The groups were randomized such that individual hearts from all 3 experimental groups were performed on multiple days. The investigator was not blinded for the experimental groups in the whole-heart perfusion studies because we were using tetrodotoxin in several experiments where safe handling is required. The concentration range of SGLT2is used in the current study was based on steady-state plasma levels observed clinically.^61,62^ In all experiments, drugs were added 20 minutes before ischemia and were present in the heart throughout the reperfusion period. After 40 minutes of reperfusion, hearts were flash-frozen and stored at −80°C.

### Immunoblot Analysis

Briefly, hearts were snap-frozen in liquid nitrogen, subsequently homogenized, and prepared according to previously reported methods.^59,60^ Protein (30–50 µg) was resolved by electrophoresis on (10%–15%) sodium-dodecyl sulfate-polyacrylamide gels and transferred onto a membrane that was probed with antibodies to GAPDH (Cat No. 5174, 1:1000; Cell Signaling Technology, Inc, MA), NLRP3 (Cat No. ab214185, 1:500; Abcam, Burlingame, CA), and TXNIP (Cat No. K0205-3, 1:500, MBL International Co, Woburn, MA). After washing, membranes were incubated with the corresponding secondary antibodies (1:5000). The blots were visualized with enhanced chemiluminescent reagent, and densitometric analysis was performed using ImageJ software (National Institutes of Health, Bethesda, MD).

### Statistical Analysis

All values represented are mean ± SEM unless stated differently. Significances are indicated as NS *P*>0.05, *P<0.05, **P<0.01, and ***P<0.001. Where possible, we collected electrophysiological and imaging data with the control and experimental test conditions in the same cell and compared using a paired *t* test, after confirming normal distribution of the data with a Shapiro-Wilk normality test. In some experiments, it was not possible to obtain vehicle control and test condition measurements in the same cell, and we therefore used an unpaired *t* test. The Western blot data were analyzed using 1-way ANOVA and Fisher least significant difference test for post hoc pairwise comparisons with α = 0.05 for each comparison. The LVDP over the different treatment groups (postischemia) was compared with a 2-way repeated-measures ANOVA with a Tukey post hoc test, and the percentage recovery of the LVDP was compared with a 1-way ANOVA and Fisher post hoc test. The indicated significances are the results of the pairwise comparisons between groups. Excel and OriginPro 9 software were used for statistical analysis.

## Results

### Empagliflozin Inhibits Late-*I*_Na_ in a Mouse Model of Heart Failure and in Sodium Channels Carrying LQT3 Mutations

**Figure 1.**
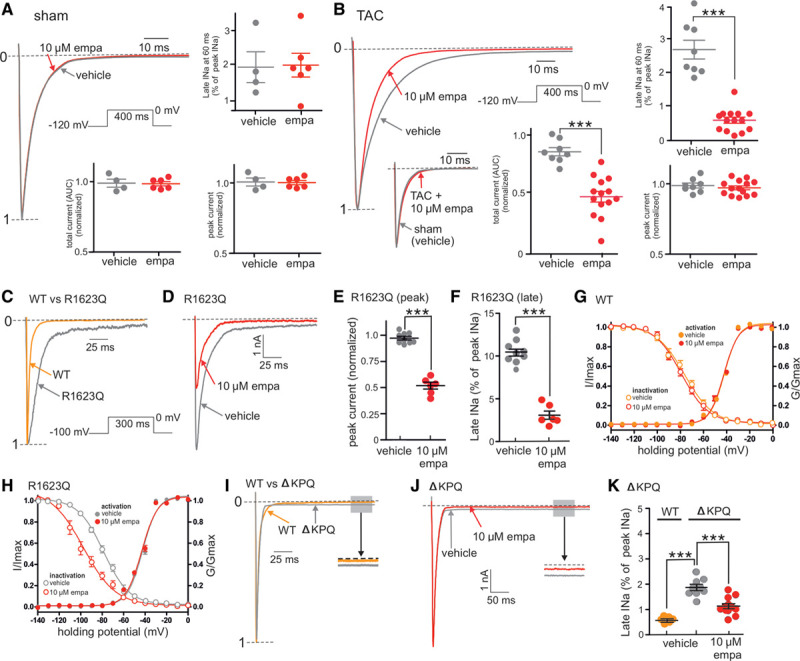
**Empagliflozin inhibits the late component of the cardiac sodium channel current (late-*I*_Na_) in heart failure and long QT syndrome 3.** **A**, Representative whole-cell patch-clamp recording and grouped data showing the lack of effect of empagliflozin (10 µmol/L, empa) on peak, late, or total sodium currents from single cardiomyocytes isolated from sham-operated hearts. **B**, Representative whole-cell patch-clamp recording and grouped data showing the effects of empa (10 µmol/L) on sodium currents from single cardiomyocytes isolated from transverse aortic constriction–operated hearts in heart failure. Grouped data are from 5 to 15 cardiomyocytes per group. AUC indicates area under the curve. ***P<0.001, unpaired *t* test. **C**, Representative whole-cell patch-clamp recordings of recombinant Nav1.5 channel sodium currents from either wild-type (WT) or the long QT syndrome 3 (LQT3) R1623Q mutant. **D**, Representative whole-cell patch-clamp recordings showing the effects of empa (10 µmol/L) on sodium currents from the R1623Q mutant Nav1.5 channel. **E** and **F**, Grouped data showing the inhibitory effects of empa on peak- and late-*I*_Na_, respectively, in the R1623Q mutant. **G** and **H**, Grouped data showing the effects of empa (10 µmol/L) on the activation and inactivation curves from WT (**G**) and R1623Q mutant Nav1.5 channels (**H**). **I**, Representative whole-cell patch-clamp recordings of recombinant Nav1.5 channel sodium currents from either WT or the LQT3 ΔKPQ mutant Nav1.5 channel. **J**, Representative whole-cell patch-clamp recordings showing the effects of empa (10 µmol/L) on sodium currents from the ΔKPQ mutant Nav1.5 channel. **K**, Grouped data showing magnitude of late-*I*_Na_ in WT and ΔKPQ mutant and the effects of empa (10 µmol/L) on sodium currents from the ΔKPQ mutant. Grouped data in **E** through **H** and **K** are from 6 to 9 cell recordings per group. **E** and **F**, ***P<0.001, paired *t* test. **K**, WT vs ΔKPQ mutant; ***P<0.001, unpaired *t* test. ΔKPQ, empa vs vehicle; ***P<0.001, paired *t* test. Currents in **A** through **D** and **I** were normalized to peak current (denoted by 1-----). G/Gmax indicates test conductance/maximum conductance; I/Imax, test current/maximum current; and peak *I*_Na_, maximum peak sodium current amplitude.

Because late-*I*_Na_ is induced in heart failure and is likely involved in the etiology of this disease,^21,29,30^ we tested the effects of empagliflozin on the cardiac sodium current in a murine model of heart failure. Cardiomyocytes were isolated either from mice that had undergone TAC to induce heart failure or from sham-operated mice. The development of heart failure was confirmed in the TAC animals via echocardiography. Sodium currents were then measured from isolated single cardiomyocytes using the dialyzed whole-cell patch-clamp technique. Application of 10 µmol/L empagliflozin to sham cardiomyocytes did not significantly alter the peak current, the total sodium current, or late-*I*_Na_ (Figure 1A). The sodium current recorded from TAC cardiomyocytes displayed a slowed inactivation and an increased late-*I*_Na_ (Figure 1B) compared with currents from sham cardiomyocytes (Figure 1A). Application of 10 µmol/L empagliflozin did not affect peak current but significantly reduced both the total current (area under the curve) and late-*I*_Na_ to values similar to those observed in sham cardiomyocytes (Figure 1B). These results demonstrate that micromolar levels of empagliflozin can inhibit the heart failure–induced dysfunction of the cardiac sodium current.

Congenital LQT3 mutations in the major cardiac sodium channel isoform Nav1.5 can alter the inactivation process and induce late-*I*_Na_ in humans, resulting in prolongation of the action potential and the ECG QT interval. These Nav1.5 LQT3 mutations increase the risk of torsades de pointes ventricular fibrillation and sudden cardiac death.^39,40^ Therefore, we tested the effects of empagliflozin on 2 well-characterized Nav1.5 LQT3 mutations, R1623Q and ΔKPQ. Recombinant Nav1.5 sodium currents containing the R1623Q mutation displayed a slowed inactivation process as well as induction of late-*I*_Na_ compared with wild-type (WT) currents (Figure 1C). Application of 10 µmol/L empagliflozin significantly reduced both the peak and late components of the R1623Q currents (Figure 1D–1F). To investigate this inhibitory effect of empagliflozin on peak R1623Q sodium current further, we generated activation/inactivation curves for WT and R1623Q currents. In WT currents, application of 10 µmol/L empagliflozin did not alter either the activation or inactivation kinetics (Figure 1G). WT half-activation voltages (V_50s_) before and after empagliflozin were –41.9 and –42.4 mV, respectively. V_50s_ for the WT before and after empagliflozin were –76.3 and –79.0 mV, respectively. In R1623Q currents, the activation and inactivation V_50s_ were similar to WT currents (Figure 1H, –42.4 and –78.7 mV, respectively). Although application of 10 µmol/L empagliflozin did not alter the activation V_50_ in R1623Q currents compared with WT (–43.3 mV), empagliflozin caused a negative shift in the inactivation V_50_ in R1623Q currents compared with WT (–97.5 mV, Figure 1H), a value that is close to the holding potential of –100 mV used in these experiments. These results indicate that, in the presence of empagliflozin, R1623Q currents are unable to fully recover from inactivation on repolarization to negative potentials, and this negative shift in inactivation likely underlies the empagliflozin-induced reduction in peak *I*_Na_ in R1623Q (Figure 1D and 1E). The second LQT3 mutant tested was the triple KPQ amino acid deletion ΔKPQ. Currents from ΔKPQ channels exhibited relatively normal fast inactivation but displayed a pronounced long-lasting steady-state persistent late-*I*_Na_ which was ≈2% of the peak current amplitude compared with late-*I*_Na_ in WT currents (≈0.5% of peak current amplitude, Figure 1I and 1K). In contrast to R1623Q currents, application of 10 µmol/L empagliflozin to ΔKPQ currents did not inhibit peak current (data not shown) but significantly reduced the steady-state late-*I*_Na_ current from ≈2% to ≈1% of peak current amplitude (Figure 1J and 1K). Taken together, these results indicate that empagliflozin is able to inhibit late-*I*_Na_ in several human LQT3 mutations that increase the risk of life-threatening cardiac arrhythmias.

### SGLT2is Are Potent Inhibitors of Late-*I*_Na_

**Figure 2.**
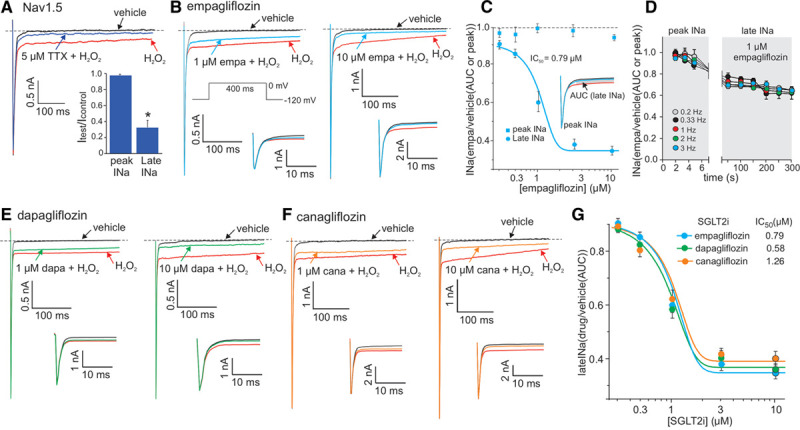
**The SGLT2 inhibitors are potent inhibitors of the late component of the cardiac sodium channel current (late-*I*_Na_).** **A** and **B**, Representative whole-cell patch-clamp recordings of H_2_O_2_-induced late-*I*_Na_ and the inhibitory effects of 5 µmol/L tetrodotoxin (TTX, **A**) and 1 or 10 µmol/L empagliflozin (empa, **B**). **C**, Concentration-inhibition curves for empa on peak- and late-*I*_Na_. **D**, Grouped data showing the frequency-dependent relationship of peak- vs late-*I*_Na_ inhibition by 1 µmol/L empa. **E** and **F**, Representative whole-cell patch-clamp recordings of H_2_O_2_-induced late-*I*_Na_ and the inhibitory effects of 1 or 10 µmol/L dapagliflozin (dapa, **E**) and canagliflozin (cana, **F**). **G**, Concentration–late-*I*_Na_ inhibition curves for empagliflozin, dapagliflozin, and canagliflozin. For all experiments, n=5 to 7 cells per group, *P<0.05 compared with the vehicle control, paired *t* test. Insets in **B**, **E**, and **F** are expanded sections illustrating the lack of effect of these drugs on peak-*I*_Na_. AUC indicates area under the curve; IC_50_, half-maximal inhibitory concentration; and peak *I*_Na_, maximum peak sodium current amplitude.

The above results from heart failure cardiomyocytes and the R1623Q and ΔKPQ Nav1.5 LQT3 mutants indicate that empagliflozin is able to inhibit late-*I*_Na_. Therefore, we performed additional experiments to further characterize the pharmacology of this interaction. Whole-cell patch-clamp recordings of peak- and late-*I*_Na_ were made from cells expressing the recombinant human Nav1.5 α-subunit. Late-*I*_Na_ was induced by application of hydrogen peroxide (H_2_O_2_, Figure 2A). Free radicals such as H_2_O_2_ can induce late-*I*_Na_ through a protein kinase C-dependent mechanism, and this pathway may contribute to the induction of late-*I*_Na_ in cardiac disease.^63^ Therefore, H_2_O_2_ was used as a tool to acutely induce late-*I*_Na_ in our model system because it also permits the recording of sodium currents before induction of late-*I*_Na_, providing an internal control for each experiment. Compared with other Nav isoforms, the peak-*I*_Na_ of the major cardiac sodium channel isoform, Nav1.5, is known to be resistant to tetrodotoxin. However, the late-*I*_Na_ component of Nav1.5 remains sensitive to micromolar levels of tetrodotoxin. Therefore, we confirmed that the H_2_O_2_-mediated induction of a persistent inward current was sensitive to 5 µmol/L tetrodotoxin (Figure 2A), indicating that this current was late-*I*_Na_.

Application of 1 and 10 µmol/L empagliflozin significantly inhibited the H_2_O_2_-induced late-*I*_Na_ (Figure 2B) in a concentration-dependent manner with a half maximal inhibitory concentration (IC_50_) of 0.79 µmol/L (Figure 2C). As selectivity for late-*I*_Na_ over peak-*I*_Na_ inhibition is a desirable pharmacological property, we also characterized the concentration-dependent effects of empagliflozin on peak-*I*_Na_. These results show that empagliflozin had little effect on peak-*I*_Na_ over a range of concentrations (Figure 2C).

As the frequency-dependent inhibition of *I*_Na_ is a common feature of many antiarrhythmic and local anesthetic drugs that act on sodium channels, we tested whether empagliflozin also displays this property. However, no significant differences in the magnitude of peak- and late-*I*_Na_ inhibition were observed on application of empagliflozin at any of the stimulation frequencies tested (0.2–3 Hz, Figure 2D).

As robust protection from heart failure is also reported with other SGLT2is, indicating a class effect, we sought to establish whether the effect on late-*I*_Na_ is unique to empagliflozin or is also observed with 2 other SGLT2is: dapagliflozin and canagliflozin. Both of these SGLT2is also inhibited late-*I*_Na_ in a concentration-dependent manner with little inhibitory effect on peak-*I*_Na_ (Figure 2E and 2F). We then performed additional experiments to generate concentration-inhibition curves for these SGLT2is (Figure 2D). Dapagliflozin and canagliflozin displayed similar concentration-dependent inhibition of late-*I*_Na_ to empagliflozin with IC_50_ values of 0.58 and 1.26 µM, respectively (Figure 2G).

### Molecular Dynamic Simulations of the Empagliflozin Binding Site in Nav1.5

**Figure 3.**
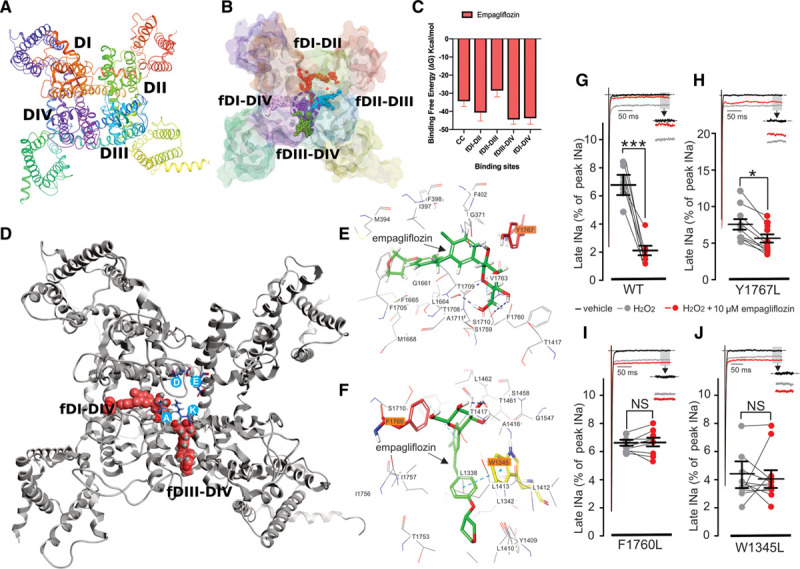
**Molecular modeling of empagliflozin (empa) docking to Nav1.5.** **A**, Macroscopic structure of the human Nav1.5 homology model used in this study. **B**, Sites chosen for focused docking shown as colored spheres, and dotted circle denotes the central cavity region. **C**, Molecular Mechanics Generalized Born Solvation Area binding free energies of empa in these different sites presented in Kcal/mol. Error bars, SD. **D**, Empa structure (crimson) docked to the fDI-DIV and fDIII-DIV sites; Selectivity filter residues are shown in blue circles (D372, E898, K1419, and A1711). **E** and **F**, Two-dimensional interaction sketches illustrating the predicted interactions of empa with residues located in the fDI-DIV (**E**) and fDIII-DIV (**F**) sites. Residues highlighted in orange are predicted to have significant interactions with the empa molecule (Y1767 in fDI-DIV, and F1760 and W1345 in fDIII-DIV). **G** through **J**, Representative whole-cell patch-clamp recordings showing the effects of empa (10 µmol/L) on sodium currents from wild-type (WT; **G**) and Y1767L (**H**), F1760L (**I**), and W1345L (**J**) mutant Nav1.5 channels. Representative current traces in **G** through **J** were normalized to peak-*I*_Na_. ***P<0.001, *P<0.05, paired *t* test. Peak *I*_Na_ indicates maximum peak sodium current amplitude.

Since the first publication of a voltage-gated sodium channel structure,^64^ the cryo-EM structures of several human sodium channels have been resolved.^44,48^ Therefore, using a homology-modeling approach on the basis of on the cryo-EM structure of human Nav1.4,^44^ we constructed a transmembrane model of hNav1.5 channel in the open state (Figure 3A). The stereochemical and local model qualities of our hNav1.5 model were also compared with the recently published cryo-EM structures of the cardiac Nav1.5^48^ and revealed a high degree of structural homology, thus validating our structural homology model (Figures I and II in the Data Supplement). Initial molecular docking of empagliflozin into all 9 plausible binding sites (named S1–S9) within Nav1.5 revealed that empagliflozin had higher binding affinities toward the 3 S1 to S3 sites, with docking scores of ≈3 Kcal/mol lower than those observed in the remaining S4 to S9 sites. The S1 to S3 sites covered multiple regions, including the central pore and the surrounding fenestration sites between the 4 domains (DI–DIV) in the Nav1.5 model. Therefore, in the next step, 5e smaller and focused grids (Figure 3B) centered on the central cavity and 4 fenestration sites were generated, named fDI-DII, fDII-DIII, fDIII-DIV, and fDI-DIV, between the DI-DII, DII-DIII, DIII-DIV, and DIV-DI domains, respectively. Empagliflozin was docked into these grids, and the best-scoring pose of the empagliflozin-Nav1.5 complex from each site was embedded within a lipid bilayer and equilibrated using MD simulations. The Molecular Mechanics Generalized Born Solvation Area binding free energies for each of these complexes were calculated from the MD snapshots (Figure 3C). The results showed that empagliflozin is predicted to preferably bind to 2 fenestration sites, namely fDIII-DIV (–44.42±2.65 Kcal/mol) and fDI-DIV (–43.79±3.27 Kcal/mol). In both sites, empagliflozin was bound in a similar orientation, where the glucose moiety of empagliflozin was located beneath the selectivity filter and facing the channel pore (Figure 3D). This binding mode in both of the sites was stabilized through the nonbonded interactions between empagliflozin and the hydrophobic residues that line the fenestration sites (Figure 3E and 3F). In addition, the glucose moiety in empagliflozin was found to be involved in many hydrogen bond interactions with residues from S6 and the pore helices (Figure 3E and 3F) as well as several water-mediated interactions.

Previous studies have shown that several sodium channel inhibitors, such as lidocaine, QX-314, benzocaine, etidocaine, and ranolazine, bind to the fDIII-DIV site within the sodium channel.^65–68^ Specifically, the amino acid F1760 is thought to be a critical interacting residue with local anesthetics and ranolazine.^65,69^ Our docking results predict that empagliflozin may also interact with F1760 as well as W1345 in the fDIII-DIV site (Figure 3F) and Y1767 in the fDI-DIV site. Therefore, to provide further information on the specific empagliflozin interacting residues, we generated separate point mutations in these 3 residues where the aromatic Y1767, F1760, and W1345 residues were substituted with a hydrophobic leucine residue to generate the Nav1.5 Y1767L, F1760L, and W1345L mutants. Currents from these mutant channels were then recorded, and late-*I*_Na_ was induced by the addition of H_2_O_2._ (Figure 3G–3J). As expected, application of H_2_O_2_ robustly induced late-*I*_Na_ in WT channels, which was significantly inhibited by 10 µmol/L empagliflozin (Figure 3G). In Y1767L mutant channels, the H_2_O_2_-induced late-*I*_Na_ was also significantly inhibited by 10 µmol/L empagliflozin (Figure 3H), although not to the same extent observed in WT channels. In contrast, the H_2_O_2_-induced late-*I*_Na_ in the F1760L and W1345L mutants was insensitive to application of 10 µmol/L empagliflozin (Figure 3I and 3J). Although we cannot discount some role for Y1767 in empagliflozin binding, our results suggest that F1760 and W1345 likely play a more critical role in empagliflozin binding to Nav1.5, indicating that the major empagliflozin binding site likely resides in the fDIII-DIV binding pocket (Figure 3F) rather than the fDI-DIV pocket (Figure 3E). These results also provide further support for the previous findings that F1760 is an important residue involved in drug binding to Nav1.5.^65,69^ Because our results show that dapagliflozin and canagliflozin exhibit similar late-*I*_Na_ inhibitory properties to empagliflozin (Figure 2E–2G), the binding poses for dapagliflozin and canagliflozin in the DIII-DIV region were also modeled (Figure III in the Data Supplement) and show good agreement with the position of empagliflozin within the putative DIII-DIV binding pocket (Figure 3F; Figure II in the Data Supplement).

### Empagliflozin Reduces Late-*I*_Na_–Induced Calcium Handling Dysfunction in Cardiomyocytes

**Figure 4.**
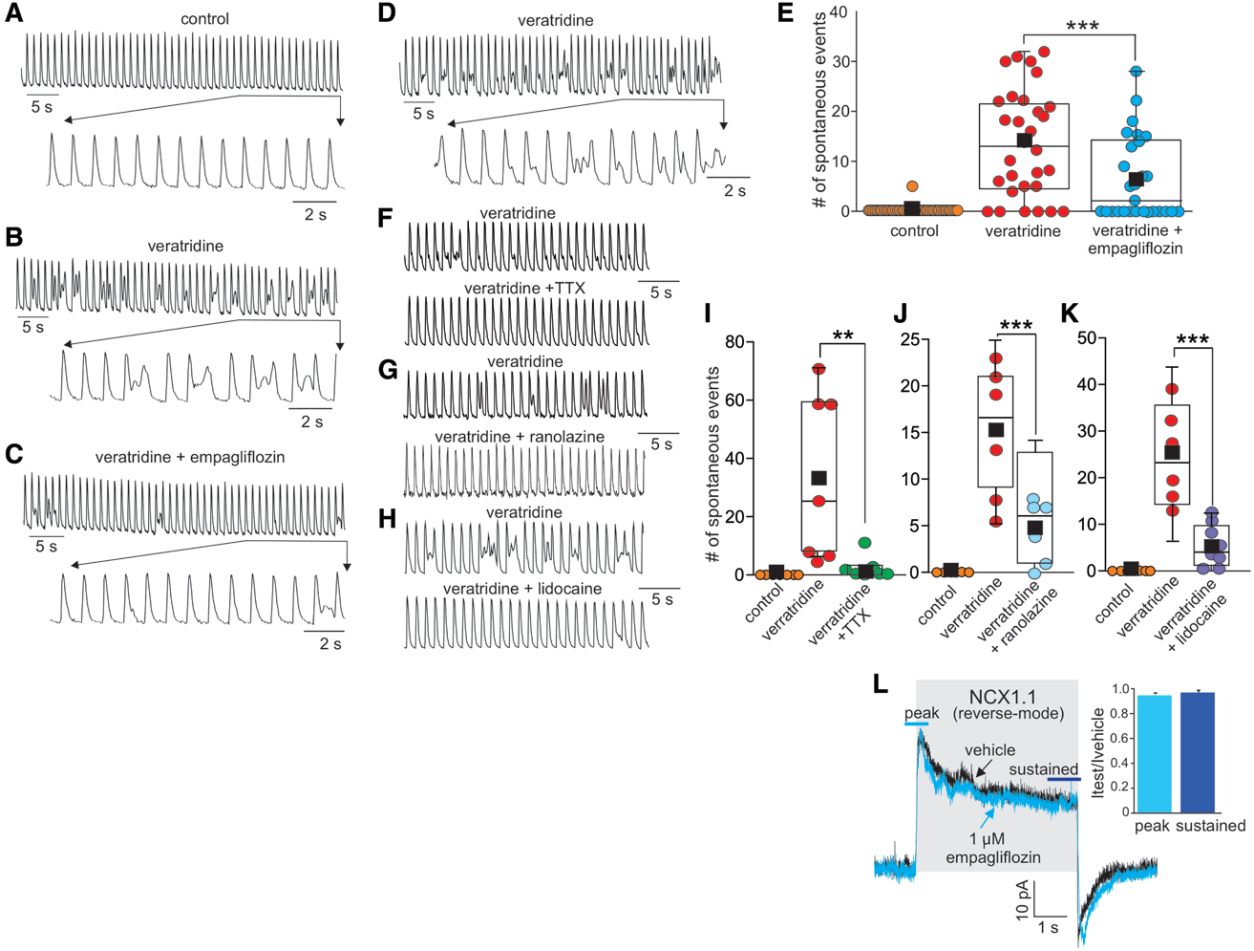
**Empagliflozin improves cardiomyocyte calcium handling induced by the late component of the cardiac sodium channel current (late-*I*_Na_) activation.** **A** through **D**, Representative traces showing that empagliflozin reversibly reduces the incidence of spontaneous calcium events induced by the late-*I*_Na_ activator veratridine. Representative 50-s sections of calcium transient recordings (1 Hz field stimulation) observed in the same myocyte under (**A**) control conditions, (**B**) during the application of 20 nmol/L veratridine, (**C**) with veratridine and 1 µmol/L empagliflozin, and (**D**) with veratridine alone after washout of empagliflozin. **F** through **H**, Representative traces of calcium transient recordings from myocytes (1 Hz field stimulation) showing that tetrodotoxin (TTX; 5 µmol/L), ranolazine (10 µmol/L) and lidocaine (50 µmol/L) also reduce the incidence of veratridine-induced spontaneous calcium events. **E**, Box plot of grouped data indicating the number of spontaneous calcium transients observed in 50-s segments in control conditions, during application of veratridine, or during application of veratridine and empagliflozin (n=28 myocytes from 7 heart isolations). **I** through **K**, Box plots of grouped data indicating the number of spontaneous calcium events either in control conditions, during application of veratridine alone or veratridine plus TTX (**I**), ranolazine (**J**), or lidocaine (**K**). n=6 or 7 myocytes per group from 5 heart isolations. **L**, Representative whole-cell patch-clamp recording and grouped data (n=5 cells) showing the lack of reverse-mode NCX1.1 inhibition by empagliflozin (NCX1.1 = sodium/calcium exchanger isoform 1.1). ***P<0.001, **P<0.01, paired *t* test.

Our results so far indicate that empagliflozin is a selective inhibitor of late-*I*_Na_ in a heart failure model and in recombinant Nav1.5 channels. However, evidence for the importance of this observation to excitation-contraction coupling and calcium handling within intact cardiomyocytes is required. Accordingly, we tested the effects of empagliflozin on calcium handling in cardiomyocytes isolated from healthy mice, where late-*I*_Na_ was induced with veratridine (Figure 4A and 4B). Veratridine was chosen to induce late-*I*_Na_ because this alkaloid specifically induces late-*I*_Na_, whereas free-radicals such as H_2_O_2_ also affect many of the cellular pathways involved in cardiomyocyte excitation-contraction coupling independent of late-*I*_Na_ induction as well as causing irreversible damage and cell death. Pilot experiments verified that 20 nmol/L veratridine induces late-*I*_Na_ activity and potentiates calcium influx, leading to the appearance of spontaneous calcium transients in cardiomyocytes (Figure 4A and 4B) without leading to irreversible calcium overload and hypercontracture that occur at higher concentrations of veratridine. Application of empagliflozin had no observable effect on the calcium transients in vehicle control-treated cardiomyocytes (data not shown). However, empagliflozin (1 µmol/L) significantly reduced the occurrence of veratridine-induced spontaneous transients by 46% (Figure 4B, 4C, and 4E). Removal of empagliflozin restores the veratridine-induced dysfunction (Figure 4D), illustrating that the effect of empagliflozin is rapid and reversible. As positive controls for our experimental system, we also confirmed that the sodium channel inhibitors tetrodotoxin, ranalozine, and lidocaine significantly reduced the incidence of veratridine-induced spontaneous transients (Figure 4F–4K). Because sodium loading resulting from the induction of late-*I*_Na_ leads to increased reverse-mode sodium/calcium exchanger activity (NCX1.1), the possibility remained that empagliflozin may reduce calcium loading via NCX1.1 inhibition. However, we observed no significant inhibition of reverse-mode NCX1.1 on the application of 1 µmol/L empagliflozin (Figure 4L).

### Empagliflozin Reduces IR Injury and NLRP3 Activation

**Figure 5.**
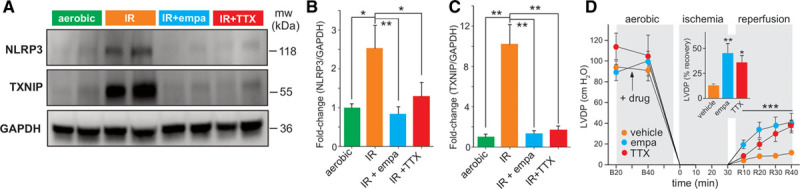
**Empagliflozin reduces NLRP3 inflammasome activation and improves postischemic contractile function.** **A**, Representative Western blot illustrating that ischemia-reperfusion of isolated mouse hearts results in the activation of NLRP3 and TXNIP, an effect that is significantly reduced by empa or the late component of the cardiac sodium channel current inhibitor TTX. **B** and **C**, Grouped data from **A** (control: n=4; empa: n=5; TTX: n=6, 1-way ANOVA). **D**, LVDP at the baseline before (B20) and after (B40) drug treatment, at ischemia, and at 10, 20, 30 and 40 minutes reperfusion (R10, R20, R30, and R40). Two-way repeated-measures ANOVA during the reperfusion phase and 1-way ANOVA in the inset. **B** through **D**, Values represent mean±SEM. ***P<0.001, **P<0.01, *P<0.05. empa indicates empagliflozin; IR, ischemia-reperfusion; LVDP, left ventricular developed pressure; mw, molecular weight; NLRP3, nucleotide-binding domain-like receptor protein 3; TTX, tetrodotoxin; and TXNIP, thioredoxin-interacting protein.

To extend these observations to a model of cardiac damage where induction of late-*I*_Na_ occurs acutely, we utilized an ex vivo IR model of cardiac injury. Furthermore, our recent research has demonstrated that empagliflozin attenuates activation of the NLRP3 inflammasome.^18^ Therefore, we investigated the role of late-*I*_Na_ in the recovery of cardiac function and activation of the NLRP3 inflammasome during IR injury. We took advantage of the fact that the peak current of the major cardiac sodium channel isoform Nav1.5 is resistant to tetrodotoxin, whereas late-*I*_Na_ is highly sensitive to tetrodotoxin (Figure 2A). Therefore, tetrodotoxin can be used as a late-*I*_Na_ inhibitor in this model system. IR injury induced an up-regulation of the NLRP3 inflammasome and its coactivator TXNIP (thioredoxin-interacting protein^70^; Figure 5A–5C). It is interesting that perfusion of the hearts with either empagliflozin or tetrodotoxin resulted in a complete prevention of the activation of the NLRP3 inflammasome and TXNIP (Figure 5A–5C). Both empagliflozin and tetrodotoxin also improved reperfusion functional recovery as determined by measurement of LVDP (Figure 5D). These results provide additional support, at the whole-heart level, for empagliflozin mediating cardioprotection in part through inhibition of late-*I*_Na_ and activation of the NLRP3 inflammasome.

## Discussion

Results from this study revealed the following key observations: (1) empagliflozin inhibits late-*I*_Na_ in a mouse model of heart failure and in Nav1.5 channels with LQT3 mutations, (2) the late-*I*_Na_ inhibitory effects of SGLT2is are a drug class effect because empagliflozin, dapagliflozin, and canagliflozin all exhibit selective inhibition of late-*I*_Na_ with comparable potency, (3) molecular simulations indicate that empagliflozin binds to a region in Nav1.5 that is a binding site for known sodium channel inhibitors, (4) empagliflozin reversibly reduced the incidence of late-*I*_Na_–induced calcium disturbances in single cardiomyocytes, and (5) empagliflozin prevented activation of the NLRP3 inflammasome and improved functional recovery in an acute model of cardiac injury in which activation late-*I*_Na_ occurs.

Induction of late-*I*_Na_ is intimately involved in the development of heart failure and cardiac arrhythmias,^21,29,30^ and drugs that inhibit late-*I*_Na_ are effective in animal models of heart failure,^32,33^ representing a novel therapeutic modality.^23,29,36–38^ Our results confirm that the cardiac sodium current inactivation process is slowed and late-*I*_Na_ is induced in cardiomyocytes from mice in heart failure. Empagliflozin significantly reduces late-*I*_Na_ and reduces sodium entry via the sodium channel to similar levels observed in sham-operated control cardiomyocytes. A previous study has shown that empagliflozin can inhibit cardiac late-*I*_Na_ in a rat model of diabetes,^41^ and we now show that heart failure–induced late-*I*_Na_ is also sensitive to empagliflozin independent of diabetes. Moreover, empagliflozin was highly selective for late-*I*_Na_ over peak *I*_Na_ in this TAC heart failure model and also did not inhibit peak-, total-, or late-*I*_Na_ in cardiomyocytes from the control sham group. Taken together, these observations indicate that empagliflozin’s effects on the cardiac sodium channel are specific to the induction of late-*I*_Na_ in disease states such as heart failure rather than a more generalized effect on the sodium current in cardiomyocytes isolated from healthy sham control animals.

Late-*I*_Na_ and slowed inactivation of the sodium current are also induced in the presence of certain Nav1.5 mutations that underlie LQT3 in humans.^39,40^ Genetic induction of late-*I*_Na_ results in action potential prolongation, an increase in the ECG QT interval, altered calcium handling, and an increased risk of developing life-threatening torsades de pointes ventricular fibrillation. Therefore, as we show that empagliflozin selectively inhibits late-*I*_Na_, we tested the effects of empagliflozin in recombinant Nav1.5 currents containing either the R1623Q or ΔKPQ LQT3 mutations. Our results demonstrate that empagliflozin also inhibits late-*I*_Na_ in channels containing these mutations. Of note is our observation that peak *I*_Na_ was also inhibited in the R1623Q mutant channel, causing a negative shift in the inactivation-voltage curve. However, empagliflozin did not inhibit peak-*I*_Na_ in ΔKPQ mutant channels. Although residue R1623 is not within the predicted binding region for empagliflozin, in the presence of this LQT3 mutation, empagliflozin may stabilize the inactivated state of Nav1.5 such that more negative voltages are required to allow recovery from inactivation, resulting in the observed reduction in peak-*I*_Na_. Further experiments on a variety of different LQT3 mutations are therefore warranted to determine whether (1) the effects of empagliflozin on peak-*I*_Na_ are specific to the R1623Q mutation, (2) empagliflozin also inhibits late-*I*_Na_ in LQT3 mutations with different locations in Nav1.5, and (3) genetic models of LQT3 are sensitive to empagliflozin and other SGLT2is. It is tempting to speculate that such experiments may also reveal whether SGLT2is could potentially be used in the future as antiarrhythmic drugs in patients harboring specific LQT3 mutations.

The location of empagliflozin binding to Nav1.5 is of obvious importance, and previous studies have mapped the interacting residues with several drugs known to inhibit the cardiac sodium current. Our computational modeling results predict that empagliflozin may bind in 2 sites within Nav1.5, the fDIII-DIV and fDI-DIV regions that line the inner pore. In particular, empagliflozin is predicted to interact with several key residues such as F1760 and W1345 in fDIII-DIV and Y1767 in fDI-IV. Some of these residues have been previously reported to bind known sodium channel inhibitors.^65–69^ For example, F1760 forms cation-pi interactions with the protonated amine group of charged class Ib antiarrhythmic drugs such as lidocaine and mexiletine,^69^ as well as interactions with the antianginal drug ranolazine.^65^ In our experiments, substitution of F1760 or W1345 with leucine eliminated the late-*I*_Na_ inhibitory effect of empagliflozin. Although our modeling data also predicted empagliflozin binding to Y1767 in fDI-DIV, the preservation of late-*I*_Na_ inhibition by empagliflozin in the Y1767L mutant argues against this residue playing critical role in empagliflozin binding. Taken together, our results provide support for empagliflozin binding to the fDIII-DIV region within Nav1.5, which is the same binding pocket for known sodium channel inhibitors.^65–69^ Future molecular and structure activity studies are warranted to determine (1) additional residues within Nav1.5 that interact with the SGLT2is empagliflozin, dapagliflozin, and canagliflozin; and (2) SGLT2i structural moieties that are crucial for late-*I*_Na_ inhibitory potency and selectivity for late- over peak-*I*_Na_. Such an approach may allow for the design of highly selective and more potent late-*I*_Na_ inhibitors that could potentially be developed for the treatment of heart failure or in patients with LQT3 mutations where the glucose-lowering effects of the SGLT2is are not required.

Our pharmacological data on recombinant Nav1.5 provides additional evidence for the SGLTis being potent inhibitors of late-*I*_Na_ because all 3 SGLTis tested (empagliflozin, dapagliflozin, and canagliflozin) selectively inhibited late-*I*_Na_ over peak-*I*_Na_ with IC_50s_ in the low micromolar range. Similar to our observations in sham cardiomyocytes, the SGLT2is had a minimal effect on the recombinant Nav1.5 currents before induction of late-*I*_Na_ with heart failure or H_2_O_2_. The use of H_2_O_2_ to induce late-*I*_Na_ in recombinant Nav1.5 channels may be criticized as being nonspecific because free radicals affect many different pathways involved in sodium and calcium homeostasis in cardiomyocytes.^19^ However, because we measured pure Nav1.5 currents in our heterologous expression system, we believe that we were able isolate the effects of H_2_O_2_ specifically to the sodium current. Moreover, we also observed late-*I*_Na_ inhibition with empagliflozin in TAC cardiomyocytes and LQT3 mutant Nav1.5 currents. Therefore, the novel effects of empagliflozin and other SGLT2is on the cardiac sodium current are unlikely specific to late-*I*_Na_ induction with H_2_O_2_ alone, but seem to be a generalized effect on late-*I*_Na_ induced by a variety of pathological or genetic mechanisms.

Our modeling data indicate that empagliflozin binds to a region of Nav1.5 that is also crucial for the pharmacological actions of local anesthetics such as lidocaine and the antianginal drug ranolazine. Because local anesthetics are classified as open-state blockers, exhibiting a frequency-dependent inhibition of the sodium current, we tested whether this may also be the case for empagliflozin. However, unlike these other known sodium channel inhibitors, we observed little frequency dependence of peak- or late-*I*_Na_ inhibition, indicating that the SGLT2is are not open-state blockers. Any potential cardioprotective or antiarrhythmic mechanisms of the SGLT2is are therefore more likely to result from selective late-*I*_Na_ inhibition and improved calcium handling rather than from a frequency-dependent inhibition of peak- and late-*I*_Na_.

Therefore, to investigate whether empagliflozin may also inhibit late-*I*_Na_ and reduce late-*I*_Na_–induced calcium handling abnormalities, we performed calcium-imaging analysis in paced single mouse cardiomyocytes. Our results demonstrate that empagliflozin, like tetrodotoxin, ranolazine, and lidocaine, drugs that are known to inhibit late-*I*_Na_ in the heart, reduces the occurrence of spontaneous calcium transients induced by the late-*I*_Na_ activator veratridine, suggesting a direct inhibitory effect of empagliflozin on late-*I*_Na_ that is rapid and reversible. However, we cannot discount any nonspecific actions of veratridine also being involved. Induction of late-*I*_Na_ causes excessive sodium loading within cardiomyocytes and the subsequent extrusion of sodium in exchange for calcium influx via an increase in reverse-mode sodium-calcium exchanger activity. Therefore, the possibility remains that empagliflozin may also reduce late-*I*_Na_ induced abnormal calcium handling in cardiomyocyte by inhibition of reverse-mode sodium-calcium exchanger activity. However, our results argue against this notion as empagliflozin did not significantly inhibit reverse-mode sodium-calcium exchanger activity in the cardiac NCX1.1 isoform. Pathophysiological conditions, such as ischemia and heart failure, also cause intracellular acidification that lead to an increase in NHE1 activity, resulting in sodium loading, induction of reverse-mode sodium-calcium exchanger activity, and excessive calcium loading. In this respect, a recent study suggests that cardiac NHE1 is a target for empagliflozin.^25,26^ Our results now provide evidence for late-*I*_Na_ also being a putative target for the SGLT2is in the heart that may contribute to the documented cardioprotective effects of this class of drug via reductions in abnormal calcium handling.

Inflammation and priming of the NLRP3 inflammasome are thought to play an important role in the development of heart failure.^71–73^ Furthermore, the SGLT2i dapagliflozin reduces NLRP3 activation and diabetic cardiomyopathy in a mouse model of type 2 diabetes.^74^ Our group has also recently shown that empagliflozin prevents worsening of heart failure in nondiabetic mice,^17^ and that empagliflozin also prevents NLRP3 activation in a calcium-dependent manner.^18^ Taken together, these results suggest that the SGLT2is have a direct suppressive effect on NLRP3 activation in the heart. This dependence on calcium suggests that the SGLT2is may be acting on an upstream target involved in calcium loading. Of particular interest is the fact that induction of late-*I*_Na_ is involved in the development of heart failure and cardiac arrhythmias.^21,29,30^ Furthermore, drugs that act in part via late-*I*_Na_ inhibition are effective in animal models of heart failure.^32,33^ Our use of tetrodotoxin as a positive control for late-*I*_Na_ inhibition demonstrates that tetrodotoxin reduced NLRP3 inflammasome activation and improved functional recovery to the same extent as empagliflozin in an acute model of cardiac injury where late-*I*_Na_ is induced and the NLRP3 inflammasome is activated. This observation also implicates a role for late-*I*_Na_ in NLRP3 priming, because the major Nav1.5 sodium channel isoform in the mouse heart displays an insensitivity to peak-I_Na_ inhibition by tetrodotoxin. Given that sodium channels are highly expressed in cardiomyocytes compared with cardiac fibroblasts, it is likely that empagliflozin inhibits late-*I*_Na_ in cardiomyocytes to contribute to the improvements observed in a model of acute IR-induced cardiac injury. However, 1 limitation of our study is that we did not test this in cardiofibroblasts, and thus we cannot rule out the possibility that cardiofibroblast inflammasome activation may also contribute to the resulting cytokine storm. We also cannot discount the possibility that empagliflozin may inhibit late-*I*_Na_ in other Nav isoforms found in the heart, for example, Nav1.2 and 1.9, which are expressed within intracardiac peripheral ganglia as well as several tetrodotoxin-sensitive neuronal isoforms that are expressed in cardiomyocytes, in addition to the major Nav1.5 isoform.

With respect to clinical implications, the calculated IC_50_ of 0.7 to 1.26 µmol/L for late-*I*_Na_ inhibition for the 3 SGLT2is tested are within the therapeutic steady-state range observed in patients.^61,62^ Therefore, significant inhibition of late-*I*_Na_ may be expected in patients prescribed SGLT2is. The protection against heart failure has been observed in numerous clinical trials with different SGLT2is,^4–8^ indicating a class effect of these drugs. Our electrophysiological data on late-*I*_Na_ inhibition from 3 different SGLT2is each indicate an IC_50_ in the low micromolar range, a finding consistent with the class effect observed in these trials.^4–8^ Of additional interest is the observation from some of these trials that the SGLT2is also reduce the incidence of sudden cardiac death, suggesting that this class of drug may reduce the occurrence of life-threatening ventricular arrhythmias. In this regard, the induction of late-*I*_Na_ contributes to action potential prolongation, calcium loading, and generation of early and delayed afterdepolarizations that provide a favorable electrophysiological substrate for the triggering of potentially fatal ventricular arrhythmias.^20,21^ Therefore, inhibition of late-*I*_Na_ by the SGLT2is may also contribute to the clinical efficacy of this class of drug against sudden cardiac death. This notion is further supported by the fact that that late-*I*_Na_ has been identified as a promising target for antiarrhythmic therapeutic intervention,^29^ and drugs that act in part through late-*I*_Na_ inhibition such as ranalozine reduce the incidence of ventricular arrhythmias.^75^ Moreover, the recent results from the EMPEROR-Reduced and DAPA-HF trials^9–11^ demonstrate that the beneficial cardiac effects of the SGLT2i dapagliflozin occur independently of diabetes, suggesting mechanisms independent of glycemic control.

Our results are consistent with a direct effect of SGLT2is on the heart, because we demonstrate a cardiac-specific and SGLT-independent mechanism in which empagliflozin’s inhibition of late-*I*_Na_ may improve cardiovascular outcomes that precede NLRP3 activation.^18^ Nevertheless, SGLT2is are also likely to mediate improved cardiovascular mortality by additional noncardiac-specific mechanisms.^8,12–16^ With respect to limitations of the models used in our study, we used ex vivo, in vitro, and molecular techniques to isolate and characterize the late-*I*_Na_ inhibitory effects of the SGLT2is, although an in vivo model of heart failure may be considered a more direct model to study the cardioprotective mechanisms of these drugs. However, it would be difficult to directly demonstrate a sole cardioprotective mechanism through inhibition of late-*I*_Na_ alone in an in vivo model, because the documented hemodynamic and metabolic effects of SGLT2is would still be present. We acknowledge that future experiments are fully warranted to further explore and dissect out the relative contribution that SGLT2i-induced late-*I*_Na_ inhibition plays in the observed cardioprotective effects of this class of drug. These studies may include (1) LQT3 genetic models, (2) ex vivo measurements of whole-heart electric activity and calcium handling in rodent models of heart failure, (3) electrophysiological measurements of late-*I*_Na_ and action potentials from human heart failure tissue samples, (4) comparison the cardioprotective and potential antiarrhythmic effects of the SGLT2is versus ranolazine or local anesthetics, and (5) examination of the structure activity relationships of the SGLT2is with respect to their putative binding site and mechanism of action of late-*I*_Na_ inhibition.

In summary, we provide evidence that late-*I*_Na_ may be a novel molecular target for the SGLT2i class of antidiabetic drug. Our structural modeling studies are based on a validated homology model of cardiac Nav1.5, providing atomic-scale mechanistic insights into how SGLT2is interact with Nav1.5 within a binding site that is common to known sodium channel inhibitors. These novel findings represent an important advance by elucidating a plausible molecular mechanism by which the SGLT2is contribute to the robust protection against heart failure and sudden cardiac death observed in numerous clinical trials to date.

## Acknowledgments

The authors thank Jody Levasseur for her expert assistance in performing the TAC surgeries. Author contributions: K.P. and S.K. designed experiments, analyzed data, generated figures, and cowrote the paper. M.F., W.L., S.S., N.J.B., A.B., J.S., T.P., C.S., A.M.D., Z.M, and J.W. performed experiments, analyzed the data, and generated figures. J.M.S. and J.R.B.D. contributed to the experimental design, project concept, and data interpretation as well as writing of the paper. K.B. provided molecular modeling expertise and oversaw the computational analysis interpretation. P.E.L. conceived the original project; contributed to all aspects of the experimental design, data analysis, interpretation of the results, and generation of the figures; and is the senior author of the paper.

## Sources of Funding

P.E.L. holds the Dr Charles A. Allard Chair in Diabetes Research. This research was supported by grants from the Canadian Institutes of Health Research (P.E.L., J.M.S., and J.R.B.D) and the Dr Rod Eidem Diabetes Research Fund (P.E.L.). K.P. is a Research Foundation Flanders (PEGASUS)^2^ Marie Skłodowska-Curie Fellow and received funding from the European Union’s Horizon 2020 research and innovation program under the Marie Skłodowska-Curie grant agreement (No. 665501) with the Research Foundation Flanders. S.S. was funded by Alberta Diabetes Institute, Alberta Innovates, and Canadian Institutes of Health Research graduate studentships. N.J.B. was funded by an Alberta Innovates graduate studentship. S.K. was supported by the Natural Sciences Engineering Research Council of Canada postdoctoral fellowship. A.M.D. is supported by an Alberta Innovates Graduate Studentship in Health Innovation and by an Izaak Walton Killam Memorial Scholarship.

## Disclosures

None.

## Supplemental Materials

Data Supplement Figures I–III

## Supplementary Material


