# Supplementary material for: Cardiac Late Sodium Channel Current Is a Molecular Target for the Sodium/Glucose Cotransporter 2 Inhibitor Empagliflozin
Source: Circulation. 2021 Jun 2;143(22):2188–204. doi: 10.1161/CIRCULATIONAHA.121.053350 (PMC8154177; doi:10.1161/CIRCULATIONAHA.121.053350)
Supplement: Supplementary file 1 [file cir-143-2188-s001.pdf]

SUPPLEMENTAL MATERIAL

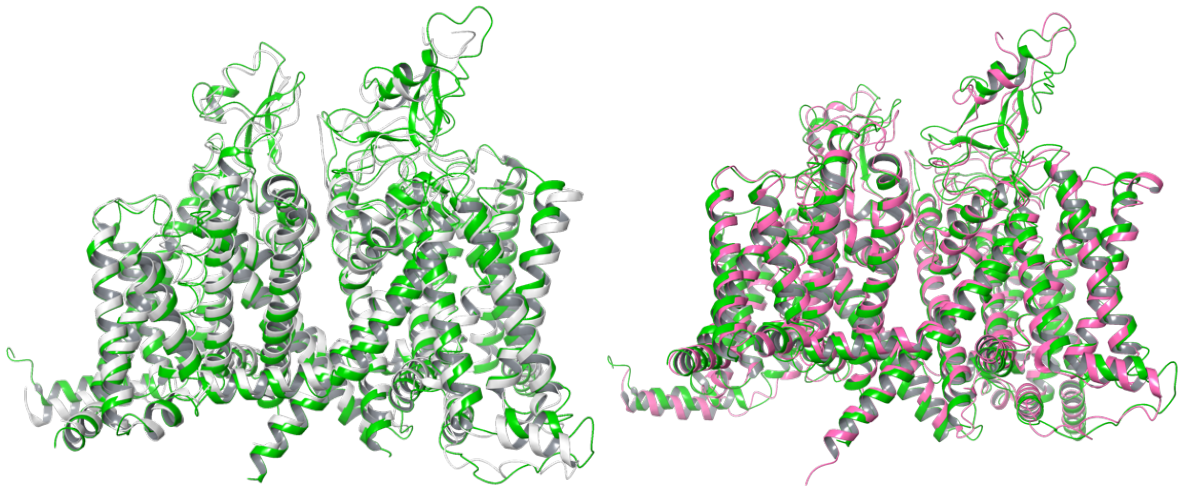

Figure SI. Structural alignments of the human Nav1.5 model used in this study with the cryo-EM structure of rat Nav1.5. 3D structure of our human Nav1.5 homology model (green ribbons) superimposed on top of the recent rat Nav1.5 Cryo-EM structures (PDB id: 6UZ3, grey ribbons, PDB id: 6UZ0, pink ribbons. The root mean square deviation values between the structures is 1.77 Å and the percentage of sequence identity between the two Nav1.5 channels is 91% (see below).

The amino acid sequence percentage identity matrix

```
1: 6UZ3_1|Chain           100.00  100.00   91.07
2: 6UZ0_1|Chain           100.00  100.00   91.07
3: sp|Q14524|SCN5A_HUMAN   91.07   91.07  100.00
```

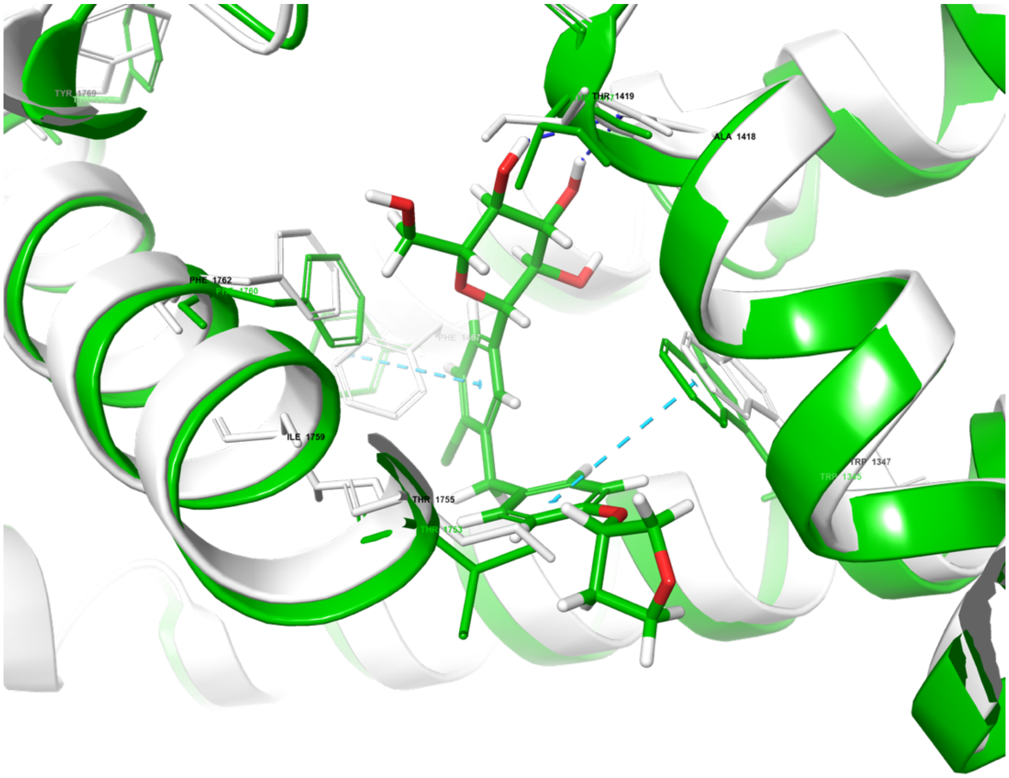

Supplementary Figure SII. Close up view of the putative empagliflozin binding pocket within the superimposed structures of our human heart Nav1.5 homology model (green ribbons) and the cryo-EM structure of the rat heart Nav1.5 model 3D (PDB id: 6UZ3, white/grey ribbons). Note that there is excellent alignment of these two Nav1.5 structures with respect to potential residue interactions with the empagliflozin molecule. Due to the slight differences in amino acid sequence between the two structures, there is a two-amino acid shift in the numbering (e.g. Phe1760 in our model = Phe1762 in the rat heart Nav1.5 structure).

A.

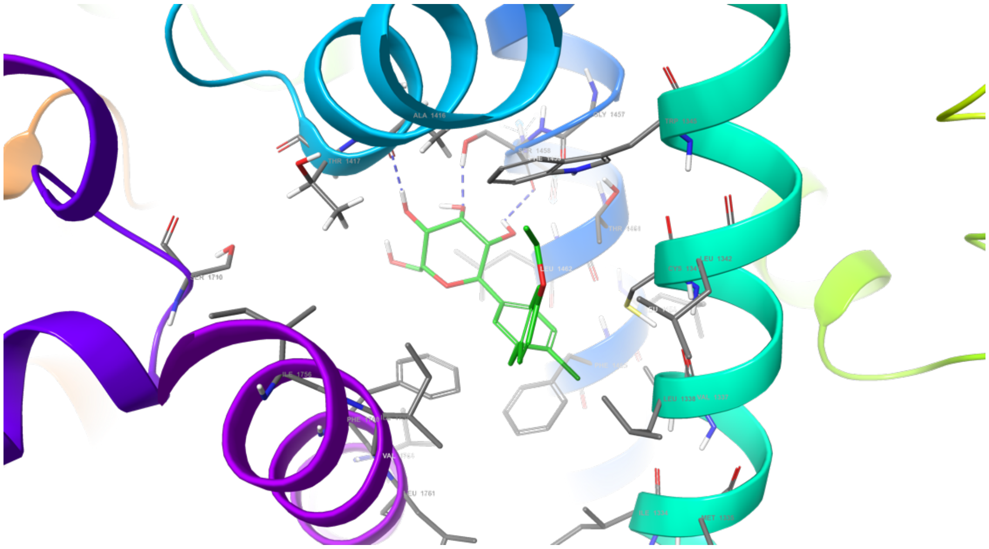

B.

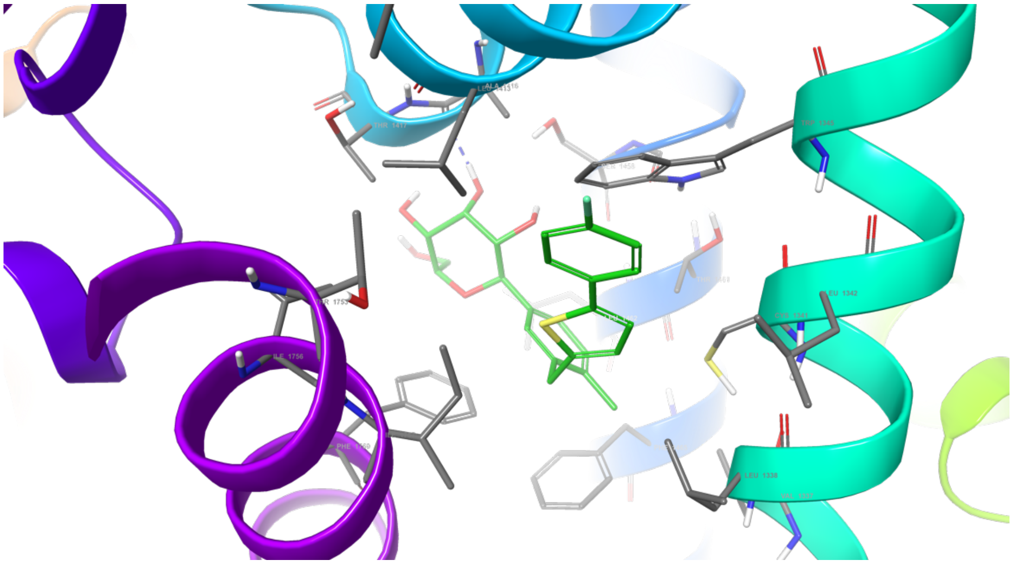

Supplementary Figure SIII. Close up view of the dapagliflozin (A) and canagliflozin (B) binding to the putative D3-D4 fenestration pocket present in the homology modelled structure of the human heart Nav1.5 channel. Note that the position and binding modes of dapagliflozin and canagliflozin structures presented in this figure resembles the binding mode observed for empagliflozin (Figures 3F and S2) in the D3-D4 fenestration site.
